# Whether surgical resection or biopsy makes difference in single lesion primary central nervous system lymphoma: a single center retrospective cohort study

**DOI:** 10.1186/s12883-022-02930-9

**Published:** 2022-11-05

**Authors:** Xin Cheng, Haoran Chen, Chongran Sun, Buyi Zhang, Jianmin Zhang, Yongjie Wang

**Affiliations:** 1 Department of Neurosurgery, School of Medicine, 2nd Affiliated Hospital, Zhejiang University, 88# Jiefang Road, Hangzhou, 310009 Zhejiang China; 2 Clinical Research Center, Neurological Diseases of Zhejiang Province, Hangzhou, China; 3 Department of Neurosurgery, International Institutes of Medicine, The Fourth Affiliated Hospital, Zhejiang University, N1 Shangcheng Road, Yiwu, 322000 Zhejiang China; 4 Department of Neurosurgery, Hangzhou Third People’s Hospital, 88# Xihu Avenue, Hangzhou, 310009 Zhejiang China; 5 Department of Pathology, School of Medicine, 2nd Affiliated Hospital, Zhejiang University, 88# Jiefang Road, Hangzhou, 310009 Zhejiang China

**Keywords:** Primary central nervous system lymphoma, Single lesion, Surgery, Biopsy, Age

## Abstract

**Background:**

Primary central nervous system lymphoma (PCNSL) is a rare and aggressive disease. The role of surgical resection in PCNSL has always been the center of debate. Here we investigated the clinical and follow-up data of single lesion PCNSL operated in our center, focusing on the comparison between surgical resection and biopsy.

**Methods:**

All consecutive cases of single lesion PCNSL between October 2004 and December 2019 were retrospectively collected from the database of the Second Affiliated Hospital of Zhejiang University, School of Medicine. Patients were divided into resection group and biopsy group. Clinical information including age, gender, Karnofsky performance status, imaging features and postoperative treatment was collected from the medical records. All the patients were followed for survival analysis.

**Results:**

A total of 105 patients with PCNSL were finally involved in our analysis. Neither PFS nor OS were significantly different between the resection group and biopsy group. The univariate analysis revealed that age < 60 and therapeutic treatment were significant predictors of longer PFS and OS. In the multivariate analysis, age (HR = 3.09, 95% CI 1.31–7.28, *p* = 0.01) and therapeutic treatment (HR = 0.25, 95% CI 0.07– 0.83, *p* = 0.02) were independent prognostic markers with OS. Multivariable Cox regression analyses also revealed that only age (HR = 2.29 (95% CI, 1.11–4.71, *p* = 0.03) was independent prognostic marker for PFS.

**Conclusions:**

In single lesion PCNSL, there was no significant difference between the resection group and biopsy group for both PFS and OS. Younger age and postoperative treatment have been proved to be indicators of better prognosis.

## Introduction

Primary central nervous system lymphoma (PCNSL) is a rare and aggressive disease, which accounts for 4% of primary central nervous system (CNS) neoplasms and 4%-6% of extranodal lymphomas. Despite advances of anti-cancer treatment modalities, the majority of the patients suffered from disease progression within the first 2 years after initial diagnosis, with a median relapse time of 10 to 18 months [1]. In a recent case series, 5-year survival rate was estimated to be 30% [2]. Once diagnosed as PCNSL, patients would receive high dose methotrexate based chemotherapy with or without autologous stem cell transplantation(ASCT) and whole brain radiotherapy(WBRT) [3]. Unlike other malignant brain tumor such as glioma and metastases, PCNSL often presents as multiple, deep-seated diffuse and infiltrative lesions, and surgical resection is controversial in extending the survival of the patient, and therefore is commonly not recommended [4]. As a result, stereotactic biopsy turns out to be the mainstream surgical intervention of PCNSL due to less invasiveness and good diagnostic accuracy.

Studies during the period of 1970s-2000s have demonstrated unfavorable complication rates and marginal benefits of surgical resection in PCNSL [5]. Indeed, De Angelis et al. even identified surgical resection as a predictor of poor outcome for PCNSL [6]. However, with the development of neuro-navigation system [7] and advancement of microsurgical techniques [8], surgical resection of PCNSL is becoming safer, more completed and better accepted. And recent studies have shown several advantages of surgical resection over biopsy. Weller et al. first demonstrated a better survival of craniotomy over that of biopsy for PCNSL [9]. Results from other studies also proved the survival benefit of surgical resection, debating a thorough rethinking of its role in PCNSL [10, 11]. Here, we investigated the association between craniotomy and survival in single lesion PCNSLs based on our single center database, together with exploration of other prognostic factors. In this study, we hope to re-valuate the role of surgical resection in PCNSLs with solitary lesion.

## Materials and methods

### Patient selection

All consecutive cases of PCNSL between October 2004 and December 2019 were retrospectively collected from the database of the Second Affiliated Hospital of Zhejiang University, School of Medicine in China. Patients all underwent either surgical resection or biopsy. Pathology was determined by an expert pathologist in all cases and based on International Classification of Diseases (ICD) code 9590/3. Patients with multifocal lesions (≥ 2 lesions), those with systemic lymphoma, those pregnant or nursing, or those for whom survival information were not available were excluded from analysis. And only patients with single lesion was included in this analysis.

### Recorded variables

Clinical information including age, gender (male vs. female), Karnofsky performance status (KPS), tumor location, size, surgical intervention and postoperative treatment was collected from the medical records. Postoperative management was defined as palliative support, and therapeutic treatment which including radiotherapy(RTx) only, chemotherapy(CTx) only and combined treatment. MRI or CT images were reviewed to record the following variables: tumor location, involvement of deep brain structures (defined as periventricular regions, basal ganglia, corpus callosum, brainstem and cerebellum), maximum visible diameter of the lesion. Patients were divided into resection group and biopsy group according to the operation records. Resection was defined as surgery with intent for maximal cytoreduction that achieved subtotal or gross-total resection, whereas biopsy was defined as the obtaining of tumor tissue for diagnostic purposes only. The surgical procedure was determined by assessment of the operative report, the neurosurgeon’s and neurooncologist’s documentation and postoperative imaging, when available. Gross total resection (GTR) was defined as removal of 95% of the tumor, while the rest was considered as subtotal resection (STR) [12, 13]. Postoperative complications were recorded and classified as systemic complication, regional complication and neurologic complications. Follow-up was conducted by means of out-of-patient clinics or telephone interview. Progression free survival (PFS) and overall survival (OS) were defined as the primary endpoints of this study. OS was determined from the date of first surgery to last follow-up or death. PFS was assessed from the date of first surgery to progression, relapse, death or last contact. The date of the last follow-up of this study was June 30, 2021.

### Statistical analysis

Analysis was performed using SPSS version 25.0 for windows. Descriptive statistics were reported as a median with a range or frequency with percentage. Survival curves were generated using the Kaplan–Meier’s methods by GraphPad Prism 8.0.2. The OS and PFS were compared by the log-rank test in the univariate analysis. All variables with *p* values less than 0.10 in the univariate analysis or variables concerned in clinical trial were involved in the multivariate analyses, using Cox proportional hazards regression model. *p* < 0.05 was confirmed as statistically significant.

## Results

A total of 105 patients with PCNSL were finally involved in our analysis. The median age was 61 years (range 17–89). The male to female ratio was 1.2:1 (57:48). 60 patients (57%) underwent resection, and 45 patients (43%) underwent biopsy. In the surgical resection group, 34(57%) patients had GTR and 26(43%) patients had STR. Only one case underwent open biopsy, and the rest cases all used method of stereotactic biopsy. The most commonly involved site was the frontal lobe (*n* = 25, 23.8%), followed by cerebellum (*n* = 18, 17.1%), corpus callosum (*n* = 12, 11.4%) and basal ganglia (*n* = 11, 10.5%). Deep brain involvement were observed in 57 patients (54.3%). 71.6% lesions were larger than 3 cm. Information about postoperative treatment was available for 77(73%) patients. Among them, only 5(6.5%) patients received palliative treatment due to low KPS, old age, economic problems or other unknown causes. 10 patients (13.0%) received radiotherapy alone, 31 patients (40.3%) received chemotherapy alone, and 31 patients (40.3%) received both radiotherapy and chemotherapy (Table 1).

**Table 1:** Baseline Patient Characteristics

	Resection (*n* = 60)	Biopsy (*n* = 45)	*P*-value
Median age/y	59 (17–76)	64 (29–89)	*P* = .004
Gender(male/female)	30/30	27/18	*P* = .309
Median KPS	70(30–90)	70(30–100)	*P* = .040
Maximum dimension			
< 3 cm	11(19.0%)	16(36.4%)	*P* = .084
≥ 3 cm	40(69.0%)	27(61.4%)	
Location			
Deep	23(38.3%)	34(75.6%)	*P* < 0.001
Superficial	37(61.7%)	11(24.4%)	
Radiation therapy			
Yes	33(55.0%)	21(46.7%)	*P* = .239
No	18(30.0%)	19(42.2%)	
Chemotherapy			
Yes	32(53.3%)	34(75.6%)	*P* = .002
No	14(23.3%)	1(2.2%)	
Median PFS/m	29.27	44.03	*P* = .567
Median OS/m	64.17	59.93	*P* = .883
Survival rate	46.7%	55.6%	*P* = .367

*Abbreviation*: *PFS*, progression-free survival, *OS * overall survival, *KPS* Karnofsky performance status;

**Fig. 1:**
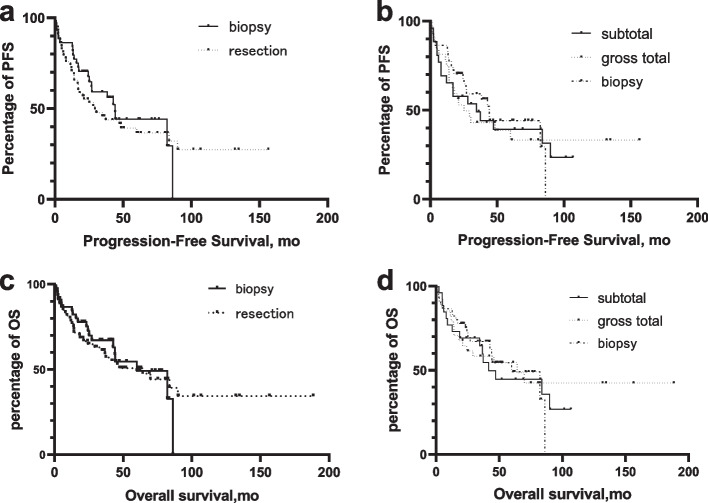
Univariate analysis performed on PFS and OS stratified by surgery types **a**. Kaplan–Meier analysis of progression-free survival of PCNSL patients stratified by biopsy versus resection. (*p* = 0.29) **b**. Kaplan–Meier analysis of progression-free survival of PCNSL patients stratified by biopsy versus subtotal resection versus gross total resection. (*p* = 0.85) **c**. Kaplan–Meier analysis of overall survival of PCNSL patients stratified by biopsy versus resection. (*p* = 0.89) **d**. Kaplan–Meier analysis of overall survival of PCNSL patients stratified by biopsy versus subtotal resection versus gross total resection. (*p* = 0.94)

Neither PFS nor OS was significantly different between the resection group and biopsy group. Median PFS of the resection group was 29.3 months (95%CI 10.9–47.6) versus 44.0 months (95%CI 35.9–52.1) of the biopsy group. Patients who underwent resection had a median OS of 64.2 months (95%CI 29.0–99.4) compared to 59.9 months (95%CI 32.6–87.2) of the biopsy group. The extent of resection also showed no significant difference for either PFS or OS. The median PFS and OS of patient who underwent STR were 34.6 months (95%CI 5.4–63.9) and 41.7 months (95%CI 27.0–56.4) respectively, while the median PFS and OS of patients with GTR were 25.2 months (95%CI 12.2–38.2) and 64.2 months (95%CI 15.3–113.0) respectively (Fig. 1).

Patients undergoing biopsy and resection had comparable rates of postoperative complications of various types. Overall, 13 patients (21.7%) in the resection group and 8 patients (17.8%) in the biopsy group experienced at least one complication. Regional complication occurred in 1 patient (1.7%) after resection and 8 patients (17.8%) after biopsy. Neurologic complication occurred in 7 patients (11.7%) after resection and 5 patients (11.1%) after biopsy. Operational related mortality happened in 1 patient, due to intracranial infection after craniotomy.

**Fig. 2:**
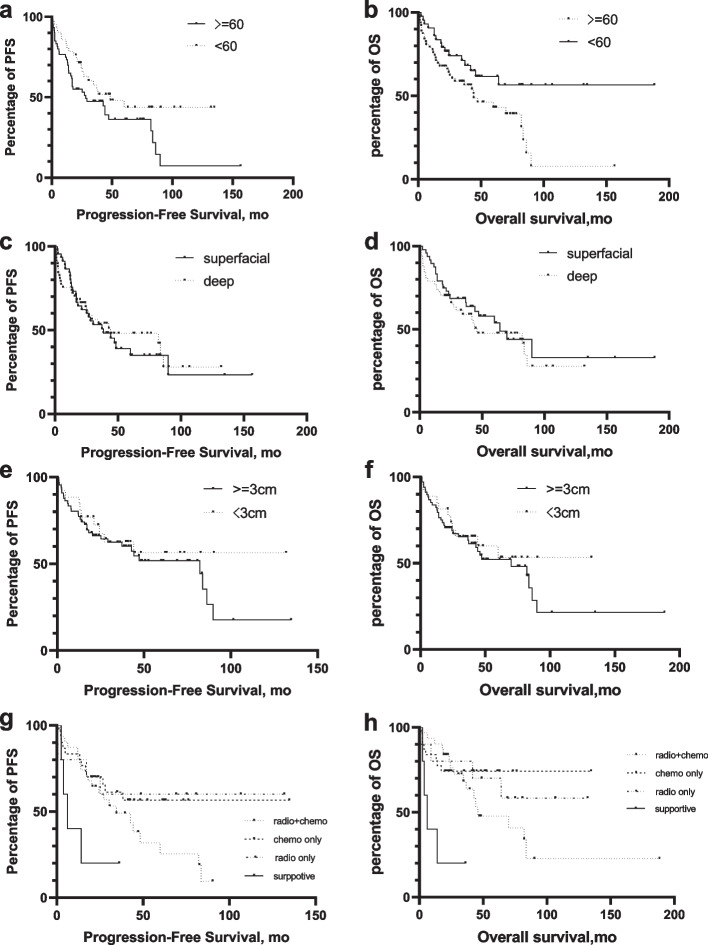
Univariate analysis performed on PFS and OS stratified by potential factors Kaplan–Meier progression-free survival curve stratified by various risk factors. Factors including age > 60 years (**a**), deep brain involvement (**c**), tumor size (**e**), MUM-1 positivity (**g**), post-operation treatment. Kaplan–Meier overall survival curve stratified by various risk factors. Factors including age > 60 years (**b**), deep brain involvement (**d**), tumor size (**f**), MUM-1 positivity (**h**), post-operation treatment

With a median follow-up of 36.2 months (range 0.6–188.3), 60 patients had experienced disease progression and 52 patients died of the disease, resulting in a median PFS of 38.4 months (95%CI 23.3–53.5) and median OS of 64.2 months (95%CI 31.5–96.8). A univariate analysis was performed for 4 potential prognostic factors including age, deep brain involvement, tumor size and postoperative treatment, which revealed that age < 60 had significantly better OS and therapeutic treatment had significantly longer PFS and better OS ( Fig. 2).

In the multivariate Cox regression analysis, age (HR = 2.98, 95% CI,1.10–8.07, *p* = 0.03) and therapeutic treatment (HR = 0.19, 95% CI 0.05–0.68, *p* = 0.01) were independent prognostic OS markers (Table 2). Multivariable analysis also revealed that only age (HR = 2.31, 95% CI 1.03–5.19, *p* = 0.04) was the independent prognostic marker of PFS.

**Table 2:** Univariable and Multivariable analysis

	Univariable analysis			Multivariable analysis		
		No	*p*-value (log-rank test)	HR	95%CI	*p*-value
Location	Deep	57	0.460	0.46	0.18,1.19	0.108
	Superficial	48				
Age	< 60	43	0.021	2.98	1.10,8.07	0.032
	≥ 60	62				
Maximum dimension	< 3 cm	27	0.440	1.57	0.56,4.37	0.390
	≥ 3 cm	68				
Surgery	Resection	60	0.883	1.10	0.40,3.06	0.848
	Biopsy	45				
Treatment	supportive	5				
	therapeutic	72		0.19	0.05,0.68	0.01
	Radio only	10	0.028	0.20	0.31,1.27	0.088
	Chemo only	31	0.010	0.14	0.31,0.59	0.008
	Radio + Chemo	31	0.001	0.22	0.57,0.84	0.026

## Discussion

PCNSL is a rare and aggressive brain malignancy with an unsatisfactory outcome associated with its complex pathogenesis and uncertain therapeutic approach. The most important risk factor for PCNSL is immunodeficiency including drug induced, infection relevant or congenital. The immunodeficiency status of patients might cause more intense side effect of treatment [14]. And the existence of the blood–brain barrier (BBB) made the delivery of chemotherapeutic drugs into the CNS more difficult, resulting in poorer outcomes of PCNSL than non-CNS lymphoma [15, 16]. Treatment of PCNSL had evolved over the last decades, but the optimal treatment regimen remains indeterminate currently. A high-dose methotrexate (HD-MTX) based multimodal therapy including other chemotherapeutic agents with or without radiation is now a consensus in the field [1]. Moreover, management of PCNSL is complex and needs an interprofessional team approach involving neurosurgeons, neurologists, oncologists, hematopathologists and radiologists [17].

Surgery is discouraged for PCNSL according to the current treatment paradigm which is based on older studies. The impactful study of DeAngelis reported a 40% complication rate associated with resection of PCNSL [6]. Both Bellinzona M. and Jahr G. showed that the impact of surgical resection on both OS and PFS was not statistically significant [18, 19]. However, recent studies highlighted the potential role of cytoreductive surgery for this disease. The results from Weller favored surgical resection and led to the reconsideration regarding the role of surgery in PCNSL [9]. Yun J. revealed that there was no difference between the complication rates following craniotomy for PCNSL and craniotomy for other CNS malignancies [20]. Studies with larger sample size by Rae A.I. reported an association between craniotomy and survival, being even more pronounced in subgroups with favorable prognostic factors [10]. Schellekes N. analyzed a cohort of 113 patients and supported surgical resection in a subgroup of PCNSL with only solitary brain lesion [11].

Our results were in accordance with previous studies on PCNSL [21] and showed that surgical resection did not improve either OS or PFS. Age and therapeutic treatment were the only positive prognostic factors for both OS and PFS, which agreed with other articles [10, 22, 23]. Both the International Extranodal Lymphoma Study Group (IELSG) [22] and the Memorial Sloan-Kettering Cancer Center scores(MSKCC) [23] considered age as an important prognostic factor, with cut-off point determined as 60 and 50 years old respectively. Our research also found that neither deep brain involvement nor tumor size had impact on PFS and OS, which differed from other studies [19, 21]. Lai R. et al. mentioned an underestimation of the tumor burden in PCNSL, caused by tumor infiltration which could only be visualized microscopically and might result in only minor radiological abnormality such as T2 hyperintensity or even completely normal appearance in MRI [24]. In 2013, Adachi K. et al. confirmed that visible contrast-enhancing lesions on MRI were only a part of PCNSL, which might be a whole-brain pathology, by reviewing the neuroimaging characteristics of PCNSL in immunocompetent patients [25]. The hypothesis might explain the limited effect of surgical resection. However, explorative craniotomy and tumor resection was still an important component of treatment paradigm. In cases of large tumor and severe mass effect, sufficient debulkation could contribute to dramatic decrease of intracranial pressure, improved neurological symptoms and better tolerance for the following chemo and radiotherapy, which might yield good results for PCNSL patients [4, 10, 26].

In this study, there was no significant differences of OS or PFS among patients receiving chemotherapy, radiotherapy or both. But the median OS of patients receiving only radiotherapy was slightly longer than those receiving combine therapy. Some researches showed that the combination of HD MTX and WBRT was associated with disabling neurotoxicity with an incidence rate of 25% to 35% and related mortality rate of 30% [27, 28]. Thiel E et al.’s phase III trial provided some evidence about the increased risk of neurotoxicity in long-term survivors brought from WBRT [29]. Moreover, Korfel A et al.’s study confirmed the late neurotoxicity. But the positive effect on PFS not OS of PCNSL brought from WBRT was also confirmed [30]. Now, In the context of HD-MTX-based chemotherapy, inclusion of rituximab during induction and thiotepa-conditioned ASCT or WBRT during consolidation are currently standard choices for PCNSL [16]. Further comparative studies are needed to validate safety and effectiveness of these therapies.

### Limitation

This study has several limitations including similar biases present in other retrospective studies. The majority of patients who underwent resection and were diagnosed with PCNSL were chosen for resection instead of a biopsy due to radiographic diagnostic ambiguity, which is not uncommon in PCNSL, combined with the surgeon’s experience and preference. And different from those typical PCNSL, these patients might show uncommon features. Thus in our analyses, the resection group had lower age, more superficial lesions. And once a patient imaging diagnosed prefer to PCNSL, our institution would more like to biopsy first followed with chemotherapy except whom was under significant mass effect and high intracranial pressure. And since patients’ condition in biopsy group would be milder, they would be more accepted to chemotherapy. Even though the size of our series was remarkable for a single-center study, the number of patients might still be inadequate to detect the real impact of surgery in this rare disease. We did not collect sufficient data of post-operational therapy. Additionally, we did not subclass the chemotherapy regimens for further survival analysis, although it was known that patients with different chemotherapy regimens tend to have different prognosis. Further multicenter prospective studies are necessary to confirm our results.

## Conclusion

In this study, we found no significant difference between the resection group and biopsy group for both PFS and OS in single lesions PCNSLs and therefore we do not advocate resection as first-line treatment. The hypothesis of considering PCNSLs as a whole-brain pathology might explain the limited effect of surgical resection. However, in single lesion PCNSL, craniotomy might play a role in cases with significant mass effect and high intracranial pressure. Further study is needed.

## Data Availability

The datasets used and analysed during the current study available from the corresponding author on reasonable request.
